# *In Silico* Prediction and Experimental Confirmation of HA Residues Conferring Enhanced Human Receptor Specificity of H5N1 Influenza A Viruses

**DOI:** 10.1038/srep11434

**Published:** 2015-06-19

**Authors:** Sonja Schmier, Ahmed Mostafa, Thomas Haarmann, Norbert Bannert, John Ziebuhr, Veljko Veljkovic, Ursula Dietrich, Stephan Pleschka

**Affiliations:** 1 Georg-Speyer-Haus, Institute for Tumor Biology and Experimental Therapy, Paul-Ehrlich-Str. 42-44, Frankfurt, Germany; 2 Institute of Medical Virology, Justus Liebig University Giessen, Schubertstrasse 81, Giessen, Germany; 3 Center of Scientific Excellence for Influenza Viruses, National Research Centre (NRC), Dokki, Giza, Egypt; 4 Robert-Koch-Institute, Division for HIV and other Retroviruses, Nordufer 20, Berlin, Germany; 5 Centre for Multidisciplinary Research, Institute of Nuclear Sciences VINCA, Mihaila Petrovica 14, Belgrade, Serbia

## Abstract

Newly emerging influenza A viruses (IAV) pose a major threat to human health by causing seasonal epidemics and/or pandemics, the latter often facilitated by the lack of pre-existing immunity in the general population. Early recognition of candidate pandemic influenza viruses (CPIV) is of crucial importance for restricting virus transmission and developing appropriate therapeutic and prophylactic strategies including effective vaccines. Often, the pandemic potential of newly emerging IAV is only fully recognized once the virus starts to spread efficiently causing serious disease in humans. Here, we used a novel phylogenetic algorithm based on the informational spectrum method (ISM) to identify potential CPIV by predicting mutations in the viral hemagglutinin (HA) gene that are likely to (differentially) affect critical interactions between the HA protein and target cells from bird and human origin, respectively. Predictions were subsequently validated by generating pseudotyped retrovirus particles and genetically engineered IAV containing these mutations and characterizing potential effects on virus entry and replication in cells expressing human and avian IAV receptors, respectively. Our data suggest that the ISM-based algorithm is suitable to identify CPIV among IAV strains that are circulating in animal hosts and thus may be a new tool for assessing pandemic risks associated with specific strains.

Influenza A viruses (IAV) have their natural reservoir in aquatic birds. However, they may acquire mutations that alter viral host tropism, leading to efficient replication in and/or transmission to other species including humans. The major potential of IAV to cause seasonal epidemics or pandemics is linked to their genomic variability. A high number of mutations results from the error-prone RNA-dependent RNA polymerase during viral RNA replication (antigenic drift). Furthermore, reassortment of genome segments may occur upon co-infection of the same cell by two or more different virus strains, often resulting in viruses with reassorted genomes and profoundly changed antigenic and biological propertie^1^,^2^,^3^. These genetic changes may cause immune escape from pre-existing neutralizing antibodies, resistance to antiviral drugs as well as changes in host tropism and/or replication efficiency in specific hosts. In addition, “candidate pandemic influenza viruses” (CPIV) may emerge from avian reservoirs and evolve into viruses that are efficiently transmitted among humans. Such pandemic strains often carry mutations in the viral hemagglutinin (HA), the virus glycoprotein that mediates binding to the cellular receptor. In some cases, amino acid (aa) substitutions in the HA cause a change in receptor usage from an avian-type receptor (α2,3-linked sialic acid, α2,3-SA) to a human-type receptor (α2,6 linked sialic acid, α2,6-SA), resulting in variants that are efficiently transmitted in humans^4^,^5^. Furthermore, aa substitutions in the HA cleavage site and/or other viral proteins may enhance the pathogenicity of newly evolved virus strains in specific hosts^6^,^7^,^8^,^9^,^10^,^11^.

Pandemic strains of IAV that encounter populations with limited pre-existing immunity may cause significant mortality and economic damage, especially if there is a delay between the identification and characterization of a new IAV strain and the production and licensing of a matching vaccine. The previous century has seen several major pandemics, including the 1918 Spanish flu (caused by an IAV H1N1 strain resulting in more than 50 million deaths), the 1957 Asian flu (H2N2, 1.5 million deaths), the 1968 Hong Kong flu (H3N2, 1 million deaths) and the 2009 Mexican flu (H1N1, 18,000 deaths)^10^,^12^,^13^. Other IAVs, such as the highly pathogenic avian influenza A viruses (HPAIV) of the H5N1 subtype, are transmitted less efficiently in humans but feature extremely high case-fatality ratios of close to 60%. These viruses were first detected in humans in 1997 in Hong Kong, where six fatalities occurred due to infection with avian H5N1-type IAV^14^,^15^. After several years with sporadic outbreaks, a second and main wave of infections with H5N1 viruses started in 2003 in some Asian countries, which then spread to Europe, the Middle East and Africa^16^,^17^. In 2006, first human infections occurred in Egypt, with sporadic infections being reported in the following years^18^. From 2003 until March 3^rd^ 2015, a total of 784 cases of human H5N1 infections were reported worldwide (292 in Egypt), leading to 429 deaths (99 in Egypt)^19^. Since 2006, clade 2.2 H5N1 viruses have evolved in Egypt by antigenic drift, resulting in distinct endemic subclades with altered virulence, pathogenicity, transmission, receptor-binding preference and drug resistance profile^20^.

Specific bioinformatic algorithms may provide tools to monitor genetic changes in circulating IAV strains, including H5N1 HPAIV, and identify IAV strains posing an increased pandemic risk, especially in geographic regions where HPAIV are endemic and frequently transmitted to humans. In this context, we recently described a novel phylogenetic algorithm based on the informational spectrum method (ISM)^21^,^22^,^23^.

In the ISM approach, sequences (protein or nucleotide) are transformed into signals by assigning a numerical value to each element (amino acid or nucleotide). These values correspond to the electron - ion interaction potential (EIIP), which determines electronic properties of amino acids and nucleotides. The signal obtained is then decomposed into a periodical function by Fourier transformation, resulting in a series of frequencies (F) and their amplitudes (A). The obtained frequencies correspond to the distribution of structural motifs with defined physico-chemical characteristics that are responsible for the biological function of the sequence. When comparing proteins that share the same biological or biochemical function(s) this technique allows the detection of code/frequency pairs that are specific for their common biological properties. The method is insensitive to the location of the motifs and, thus, does not require previous alignment of the sequences. The ISM was successfully applied to predict the biological function of novel proteins, in structure/function analyses of different protein and DNA sequences and in *de novo* design of biologically active peptides ( http://www.vin.bg.ac.rs/180/anims/ISMEIIP_Approach.html).

The calculation of the informational spectra (IS) of IAV HA aa sequences allowed us to identify minimal changes (single or combined aa substitutions) that likely affect interactions of the viral HA protein with the cellular receptor^24^. We previously used this algorithm to monitor *in silico* the human tropism of highly pathogenic H5N1 viruses isolated in Egypt^21^.

In the present study, we extended the ISM analysis to H5-HA sequences of circulating viruses to identify aa substitutions predicted to enhance the tropism to human hosts, thereby possibly affecting the respective viruses’ pandemic potential. ISM analysis of H5N1 HA1 sequences of HPAIV circulating between 2006 and 2014 in Egypt suggested that the mutations K153D, S223N and G272S may significantly increase the affinity of these viruses towards the human receptor. This is based on the observed increase in amplitude at the frequency (F) 0.236, representing the interaction with human cells, which is accompanied by a reduction in amplitude at F0.076, representing the interaction with avian cells. To validate our conclusions derived from these *in silico* studies, we generated replication-incompetent HA-pseudotyped retroviral particles (PRP) expressing H5-HA proteins with the respective codon substitutions (single or combined) and studied the entry of these PRPs into cells overexpressing human HA receptors *in vitro*. To further corroborate this set of data, recombinant IAV carrying equivalent substitutions in their HA genes were generated and their replication was analyzed in cell culture. As a safety measure, the multibasic HA-cleavage site present in the pathogenic H5-HA was replaced by a monobasic cleavage site in both the wildtype construct (Wt-LP IAV) and the mutants, resulting in viruses that required trypsin for fusion activity. Viruses carrying the specific HA substitutions were found to replicate more efficiently in mammalian cells overexpressing receptors for human HA, when compared to the parental Wt-LP IAV.

Interestingly, our analyses of H5N1 HPAIV isolates collected in Egypt over the past few years revealed a strong increase in prevalence of the predicted mutations in the HA coding sequences. Also, the predictions and hypotheses derived from our *in silico* analysis combined with our subsequent experimental studies to validate these predictions are in line with the recent natural evolution of IAV in Egypt. Thus, a fairly consistent picture arises with respect to mutations that may play a role in the adaption of avian IAV to the human receptor. The available evidence leads us to suggest that Egyptian H5N1 HPAIV are evolving towards increased human tropism, a prerequisite for increased pandemic potential of these viruses. Our study illustrates the power of bioinformatic approaches in making early predictions on possible adaptation of specific strains to new hosts and the emergence of CPIV.

## Materials and Methods

### Cells and viruses

Madin-Darby canine kidney cells (MDCK-II), human lung adenocarcinoma epithelial cells (A549), MDCK-SIAT1 cells (MDCK cells engineered to overexpress α2,6 sialic acid transferase), human rhabdomyosarcoma (TE671) cells and human embryonic kidney cells (293T) were maintained in Dulbecco’s Modified Eagle Medium (DMEM) (Gibco, Invitrogen) supplemented with 100 I.U./ml penicillin, 100 μg/ml streptomycin and 10% fetal bovine serum (FBS). Quail fibroblasts (QT6) were cultured in Ham’s F10 medium (Gibco, Invitrogen) supplemented with 100 I.U./ml penicillin, 100 μg/ml streptomycin, 2 mM L-glutamine, 10% tryptose phosphate broth, 5% chicken serum, and 10% bovine calf serum. MDCK-, 293T-, A549-, TE671- and QT6 cells were either obtained from the cell culture collection in Giessen or from LGC Promochem GmbH. MDCK-SIAT-1 cells were kindly provided by Mikhail Matrosovich, Institute of Virology, Philipps University, Marburg, Germany^25^. All cell monolayers were incubated at 37 °C in the presence of 5% CO_2_. The HPAIV isolate A/Thailand/KAN-1/2004 (KAN-1, H5N1) was kindly provided by P. Puthavathana, Thailand. Cells were infected at the indicated multiplicity of infection (MOI).

### Characterization of avian and human HA receptor expression

To determine the abundance of specific sialic acid (SA) species (SAα2,3Gal and SAα2,6Gal) on the surface of MDCK-SIAT1, QT6 and A549 cells, cell monolayers were fixed with 3% paraformaldehyde for 1 h at room temperature (RT) followed by 3 washing steps with PBS. Cells were incubated for 1 h at RT with fluorescein-labelled Sambucus nigra agglutinin (SNA, Biozol, Germany, 1:100 dilution) to detect α2,6-SA or with biotinylated Maackia amurensis lectin II (MAAII, Biozol, Germany, 1:100 dilution) to detect α2,3-SA. Binding of biotinylated MAAII was visualized using streptavidin-Cy3 (Sigma-Aldrich, Germany) at a 1:500 dilution. Cell monolayers were washed with PBS and permeabilized with 0.2% Triton X-100 for 20 min. After another washing step with PBS, nuclei were stained with DAPI (4′, 6′-diamidino-2-phenylindole; 1:400 dilution) (Roth, Germany) for 10 min at RT. Finally, cells were washed with PBS and water, embedded in Fluoromount-G (eBioscience, Germany) and analyzed by confocal laser-scanning microscopy (Leica TCS SP5).

Moreover, the presence of α2,3- and α2,6-linked SA on A549, QT6 and MDCK-SIAT1 cells was measured by FACS using different concentrations of biotinylated lectins MAAII (α2,3-SA specific) and SNA/EBL (α2,6-SA specific), respectively, and PE-conjugated streptavidin. Briefly, 10^6^ cells were incubated with 10–100 ng of the biotinylated lectins for 30 min at 4 °C. After washing in PBS, cells were stained with PE-conjugated streptavidin (1:500) for 30 min at 4 °C, washed again and PE-positive cells were quantified by flow cytometry (FACS Calibur, Becton Dickinson).

### *In silico* prediction of HA mutations with pandemic potential

For *in silico* prediction of mutations in H5-HA that might affect IAV cell tropism we analyzed the HA protein of KAN-1 by ISM, a virtual spectroscopy method for structure/function analysis of proteins^26^,^27^. ISM is based on the assumption that the protein-protein interaction encompasses two basic steps: (i) recognition and targeting between interacting proteins (long-range interactions at distances >5Ả) and (ii) chemical binding (short-range interactions at distances <5Ả)^27^. The long-range interaction properties of biological molecules are determined by the electron-ion interaction potential (EIIP) representing the main energy term of valence electrons^22^. The EIIP for organic molecules can be calculated by the following simple equation derived from the “general model pseudopotential^28^”:


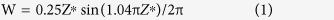


where Z* is the average quasivalence number (AQVN) determined by:


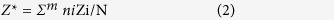


where Z_*i*_ is the valence number of the *i*-th atomic component, *n*_*i*_ is the number of atoms of the *i*-th component, *m* is the number of atomic components in the molecule, and N is the total number of atoms. Applying the given equations (1) and (2) to 20 amino acids, the following EIIP values are obtained (in Ry): L 0.0000, I 0.0000, N 0.0036, G 0.0050, V 0.0057, E 0.0058, P 0.0198, H 0.0242, K 0.0371, A 0.0373, Y 0.0516, W 0.0548, Q 0.0761, M 0,0823, S 0.0829, C 0.0829, T 0.0941, F 0.0946, R 0.0959 and D 0.1263. The ISM technique is based on a model of the primary structure of a protein using a sequence of numbers, by assigning to each amino acid the corresponding EIIP value. The obtained numerical sequence, representing the primary structure of a protein, is then subjected to a discrete Fourier transformation, which is defined as follows:


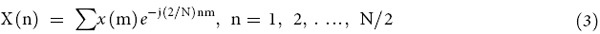


where x(m) is the m-the member of a given numerical series, N is the total number of points in this series, and X(n) are discrete Fourier transformation coefficients. These coefficients describe the amplitude, phase and frequency of sinusoids, which comprised the original signal. The absolute value of a complex discrete Fourier transformation defines the amplitude spectrum and the phase spectrum. The complete information about the original sequence is contained in both spectral functions. However, in the case of protein analysis, relevant information is presented in an energy density spectrum^23^, which is defined as follows:


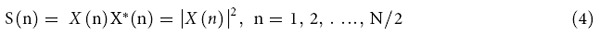


In this way, sequences are analyzed as discrete signals. It is assumed that their points are equidistant with the distance d = 1. The maximal frequency in a spectrum (as defined above) is F = 1/2d = 0.5. The frequency range is independent of the total number of points in the sequence. The total number of points in a sequence influences only the resolution of the spectrum. The resolution of the N-point sequence is 1/n. The n-th point in the spectral function corresponds to a frequency f(n) = nf = n/N. Thus, the initial information defined by the sequence of amino acids can now be presented in the form of an informational spectrum (IS), representing a series of frequencies and their amplitudes.

The IS frequencies correspond to the distribution of structural motifs with defined physicochemical properties determining a biological function of a protein. When comparing proteins that share the same biological or biochemical function, the ISM technique allows detection of code/frequency pairs that (i) are specific for their common biological properties or (ii) correlate with their specific interaction(s). These common informational characteristics of sequences are determined by the cross-spectrum or the consensus informational spectrum (CIS). A CIS of N spectra is obtained by the following equation:


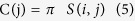


where Π(i,j) is the j-th element of the i-th power spectrum and C(j) is the j-th element of CIS. Thus, CIS is the Fourier transform of the correlation function for the spectrum. Therefore, any spectral component (frequency) that is not present in all the compared informational spectra is eliminated. Peak frequencies in CIS represent the common information encoded in the primary structure of the analyzed sequences. This information corresponds to the mutual long-range interaction between analyzed proteins or their interaction with the common interactor.

ISM analysis allows: (i) to predict the biological function of a protein; (ii) to compare the biological activity within a group of proteins with the same function; (iii) to predict mutations that may increase or decrease the biological activity of a protein; (iv) to design an artificial protein sequence with a desired biological function.

The web tool for the calculation of informational spectra of proteins is available at: http://www.vin.bg.ac.rs/180/istree/ and sequences of the different HA versions can be found in the supplementary information.

### Cloning and mutagenesis of HA

The H5-HA gene of KAN-1 was amplified by reverse RT-PCR from viral RNA isolated from virus-containing supernatants of infected MDCK cells. The HA sequence was amplified using primers Xma-HA-U (5′-TATTCCCGGGAGCAAAAGCAGGGG-3′) and Xma-HA-L (5′-ATATCCCGGGAGTAGAAACAAGGGTGTTTT-3′) and cloned via XmaI into a pHCMV-based vector as previously described^29^, resulting in M288-H5-HA. Single amino acid changes were introduced into M288-H5-HA by site-directed mutagenesis (quick change kit from Agilent Technologies). Double and triple mutations were introduced consecutively and all mutations were confirmed by sequencing.

### Generation of H5-HA-pseudotyped retroviral particles (PRP)

To generate replication-incompetent HA-PRP for single-round infection assays, we cloned (in addition to our HA constructs) the NA and M genes of KAN-1 into the M288 vector, resulting in M288-H5-NA and M288-H5-M. Primers for NA were Xma-NA-U (5′-TATTCCCGGGAGCAAAAGCAGGAGT-3′) and Xma-NA-L (5′-ATATCCCGGGAGTAGAAACAAGGAGTTTTT-3′ ) and for M Xma-M-U (5   ′-TATTCCCGGGAGCAAAAGCAGGTAG-3 ′        )  and Xma-M-L (5′-ATATCCCGGGAGTAGAAACAAGGTAGTTTTT-3  ′          ). Oncoretroviral PRP were essentially prepared as described previously^29^. Briefly, the three plasmids M288-H5-HA, M288-H5-NA and M288-H5-M (1 μg each) were cotransfected with the MLV GagPol vector (pSV-Mo-MLVgagpol^30^ M579 (12.5 μg)) and the GFP transfer vector (pMP71-eGFP-pre^31^ M56a (7.5 μg)) into 293T cells (6 × 10^6^ cells per 10 cm diameter dish) by calcium phosphate transfection. At 6 h posttransfection (p.t.), the transfection medium was replaced with DMEM containing 10% FCS, 2% L-glutamine, 100 I.U./ml penicillin, 100 μg/ml streptomycin. PRP-containing supernatants were harvested at 48 and 72 h p.t. After filtration (0.45 μm filter), PRPs were concentrated by low speed centrifugation (16 h, 6,000 rpm, 4 °C), resuspended in PBS and aliquots stored at –80 °C until further use.

### Electron microscopy of H5-HA PRP

Two days p.t., cells were fixed with 2.5% glutaraldehyde in 0.05 M Hepes (pH 7.2). Then, cells were scraped off, centrifuged, post-fixed with OsO_4_ (1%), block-stained with uranyl acetate (2%), dehydrated stepwise in graded alcohol, immersed in propylenoxide and embedded in Epon (Serva, Heidelberg) with polymerisation at 60 °C for 48 h. Ultra-thin sections (60–80 nm) were cut using an ultramicrotome (Ultracut S or UCT; Leica, Germany) and stained with 2% uranyl acetate and lead citrate. Transmission electron microscopy was performed using an EM 902 (Zeiss) operated at 80 kV. Images were digitized using a slow-scan charge-coupled-device camera (Pro Scan; Scheuring, Germany).

### Determination of receptor specificity of mutant H5-HA PRP

Mutant H5-HA PRP were titered on A549 cells, which express sialic acid receptors for human as well as avian IAV. Briefly, 5 × 10^4^ cells were seeded in the wells of a 24-well plate. The next day, cells were incubated in duplicate with dilutions (0.1–20 μl) of the appropriate mutant PRP stock. After three days, transduction rates were determined by quantifying GFP-positive cells by flow cytometry (FACS Calibur, Becton Dickinson). PRP titers were calculated as:


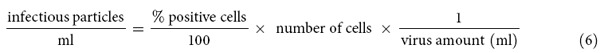


To compare potential differences in receptor selectivity of the different HA mutants, equal amounts of the respective PRPs (MOI = 0.2 as determined on A549 cells) were added to QT6 cells, which predominantly express the α2,3-SA receptor bound by avian HA, and MDCK-SIAT1 cells, which also express the avian receptor, but overexpress the α2,6-SA receptor bound by human HA^25^. Transduction rates of the two cell lines with the different mutant HA-PRPs were determined by quantifying GFP-positive cells by flow cytometry. Differences in receptor usage were determined by comparing relative numbers of GFP-positive cells determined on both cell lines (MDCK-SIAT1/QT6) using wildtype and mutant PRP, respectively.

### 3D structure model of the HA1 protein of IAV A/Thailand/1(KAN-1)/2004(H5N1)

The structural model of the H5 HA molecule of KAN-1 was constructed using SWISS-MODEL^32^ and POLYVIEW-3D^33^ and is based on homology modeling using the crystal structure of the H5-HA protein of A/Vietnam/1203/2004 (PDB ID: 2FK0.1) as a template^34^. H5 numbering was based on the mature protein (Gene bank accession: AAS65615.2) and the residues involved in receptor binding were defined according to Duvvuri *et al.*^35^.

### Generation of H5-HA IAV with a monobasic cleavage site

The reverse genetic system encoding the 8 viral segments of HPAIV strain A/Thailand/KAN-1/2004 (KAN-1, H5N1) was kindly provided by Stephan Ludwig, Muenster, Germany. To clone the H5-HA gene encoding the monobasic cleavage site, a cDNA to be used as template in subsequent PCRs was generated using the Uni-12 primer^36^. The multibasic cleavage site PQRERRRKKR/GLF between HA1 and HA2 was mutated to a monobasic cleavage site, PQRETR/GLF, using specific primers to amplify HA1: Ba-LPAI-F1 (5′-TATAGGTCTCAGGGAGCAAAAGCAGGGGTTCAATC-3′) and Ba-LPAI-R1 (5′-TATTGGTCTCTGTCTCTCTTTGAGGGCTATTTCTG-3′) and to amplify HA2: Ba-LPAI-F2 (5′-TATCGGTCTCAAGACGAGAGGATTATTTGGAGCTAT-3′) and Ba-LPAI-R2 (5′-ATATGGTCTCGTATTAGTAGAAACAAGGGTGTTTTT-3′). Briefly, 2 μl cDNA were mixed with 25 μl 2x “Phusion Master Mix” (Thermo Scientific, USA), 2 μl of each primer (40 pmoles each), and the total volume was adjusted to 50 μl using nuclease-free water (Ambion, USA). The PCR reactions were then subjected to a pre-denaturation step at 98 °C for 30 sec, followed by 35 amplification cycles (98 °C/10 sec, 58 °C/30 sec and 72 °C/1.5 min) and a final extension at 72 °C/5 min. The PCR products were purified using the “GeneJET Gel Extraction Kit” (Thermo Scientific, USA) and digested with BsaI-HF (NEB, Germany). The digested and purified PCR products were ligated with AarI-digested pMP*ccd*B DNA as previously described^37^ and transformed into *E. coli* DH5α (New England Biolabs, USA).

A part of the HA1 sequence of monobasic KAN-1/HA cloned into pMP*ccd*B was replaced with the corresponding sequences from the appropriate M288-H5-HA mutant constructs (Mut-I: S223N and G272S; Mut-II: K153D and S223N; Mut-III: K153D, S223N and G272S) using Mva1269I and EcoRI (Thermo Scientific, USA). The dual-promotor plasmids with mutated monobasic HA were transformed into competent *E. coli* XL-1 blue, screened, sequenced and then used for transfection (“NucleoBond® Xtra Maxi”; Macherey-Nagel, Germany).

### Generation of IAV stocks and determination of replication kinetics

To generate the KAN-1 (Wt-LP) virus and its mutant counterparts, 8 μg of plasmid DNA (1 μg of each plasmid) encoding the eight viral segments were transfected into a co-culture of 293T/MDCK-II cells (ratio 3:1, 10 cm^2^) as previously described with minor modifications^37^,^38^,^39^. Briefly, the transfection mixture consisting of 180 μl “Opti-MEM” (Gibco, Invitrogen, USA), 8 μg of plasmid DNA (1 μg of each plasmid), and 16 μl “Trans-IT2020” (Mirus, USA) was incubated for 45 min at RT. The transfection mixture was then diluted to 1 ml using Opti-MEM and transferred to an 80–90% confluent cell monolayer to allow transfection. The cells were then incubated for 8 h at 37 °C in the presence of 5% CO_2_. The transfection medium was replaced with 1 ml of infection medium (“Opti-MEM” containing 100 I.U./ml penicillin, 100 μg/ml streptomycin and 0.2% BSA (PAA, Germany)) and cell cultures were incubated for another 12 h. Then, an additional 1 ml of infection medium containing 2 μg/ml TPCK-treated trypsin was added (Sigma-Aldrich, USA). The cell culture supernatant was harvested 48 h after addition of the TPCK-treated trypsin and cell debris was removed by centrifugation at 2500 rpm for 5 min at 4 °C. An aliquot of 500 μl of each supernatant was used to inoculate fresh MDCK-II cells (25 cm^2^) and cultures were incubated for 72 h in the presence of TPCK-treated trypsin (1 μg/ml). Titers of recombinant viruses were determined as FFU/ml in MDCK-II cells^40^.

To determine multi-step growth curves, confluent monolayers of A549, QT6 and MDCK-SIAT1 cells, respectively, were each infected in triplicate with KAN-1 (Wt-LP) and its mutant derivatives, respectively, at an MOI of 0.001 FFU/cell. After 1 h of incubation at RT, the medium was replaced with infection medium (minimal essential medium with 100 I.U./ml penicillin, 100 μg/ml streptomycin, 0.2% BSA and 1 μg/ml TPCK-treated trypsin) and the cells were incubated at 37 °C. Supernatants were collected at 1, 24, 36 and 48 h postinfection and stored at −70 °C. Virus titers were determined by focus assay^40^.

### Biosafety

All experiments with infectious virus were performed according to German regulations for the propagation of influenza viruses. All experiments involving highly pathogenic avian influenza A viruses were performed in a biosafety level 3 (BSL3) containment laboratory approved for such use by the local authorities (RP, Giessen, Germany).

## Results

### IS frequency analysis

According to the ISM concept, interactions between biological molecules encompass two steps: (i) specific recognition by interacting molecules (long-range interactions with distances between 5 and 1000 Å) and (ii) chemical binding (short-range interactions with distances <5 Å). Without the first step the number of collisions between interacting molecules will be small, and without the second step the interacting molecules will fail to bind to each other. In conclusion, each of these two steps represents a “necessary but not sufficient” condition for efficient interaction between biological molecules measured by their “productive collision”.

As was demonstrated previously for an efficient interaction, the protein domains, which are essential for recognition and targeting (recognition and targeting site, RTS) and the receptor binding domain (RBD), should be located close to each other^41^. Specific long-range interactions between proteins are represented by the common frequency components in their informational spectrum (IS), and the strength of the interaction is represented by the amplitude values on this frequency. This common frequency is determined by a peak in the cross-spectrum of proteins (CS). The presence of a common frequency component in the IS of a group of proteins suggests that these proteins have common interactor characteristics.

Mutations that decrease the amplitude value of the IS frequency (responsible for long-range interaction between proteins) decrease the efficacy of protein interactions by decreasing the efficacy of their recognition and targeting resulting in a decreased number of productive collisions. Conversely, mutations that increase this amplitude increase the efficacy of protein-protein interactions. Therefore, mutations that affect the amplitude on a characteristic frequency, although located outside the chemical RBD, also affect the efficacy of protein-protein interactions.

We previously showed that HA1 from H5N1 HPAIV that predominantly interact with the avian receptor are characterized by the IS frequency F(0.076), whereas HA1 from viruses that preferentially infect humans are characterized by the frequency F(0.236)^41^. Thus, the amplitude values of F(0.076) and F(0.236) can be used as indicators of the respective viral tropism (bird versus human). A ratio of A(0.236)/A(0.076) <1 suggests that the virus preferentially infects birds. Conversely, A(0.236)/A(0.076) >1 suggests that the virus is more likely to infect humans. Therefore, mutations in the H5N1 HA1 that decrease the amplitude F(0.076) and/or increase the amplitude F(0.236) are predicted to improve the recognition of the human receptor, leading to a virus that, most likely, infects humans more efficiently. In other words, increased A(0.236)/A(0.076) ratios indicate that viruses expressing the respective HA1 protein are more likely to infect humans.

**Figure 1:**
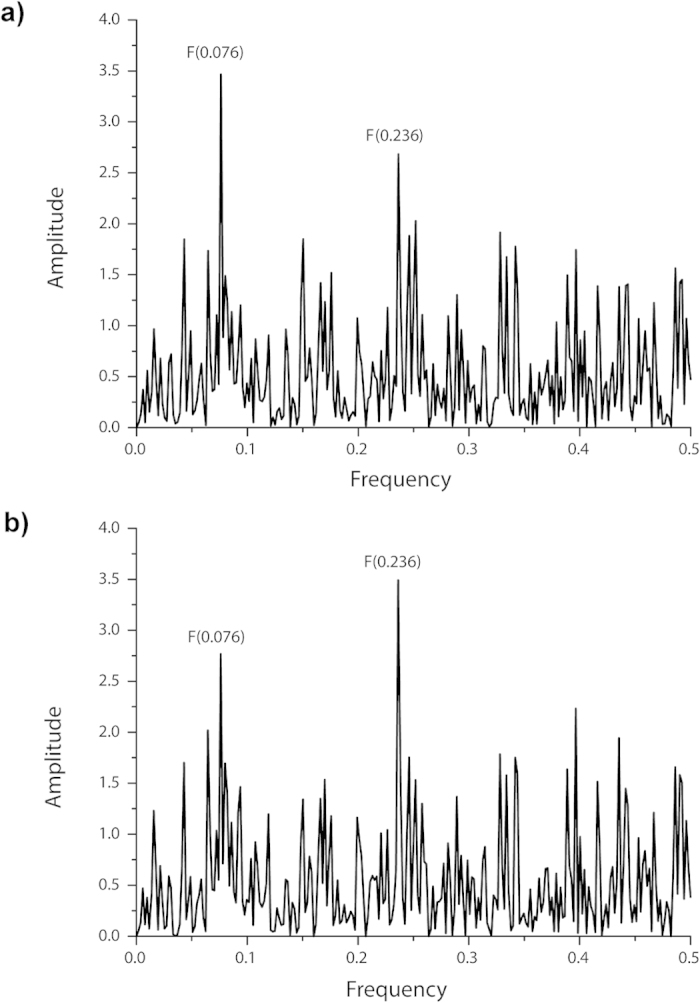
Effects of combined mutations K153D, S223N and G272S in H5-HA of A/Thailand/KAN-1/2004 (KAN-1) on the informational spectra (IS). **a**) IS of HA1 of the wildtype KAN-1 virus, **b**) IS of HA1 from the KAN-1 virus-derived triple mutant. Frequencies at 0.076 and 0.236 for avian and human receptor specificity are indicated.

In line with this, a recent analysis of mutations in H5N1 viruses circulating in Egypt between 2006 and 2012 revealed a total of 10 aa substitutions (P74S, H110R, A127T, F143Y, K153D, S188K, S223(N/I), S234P, G272S, N275S) that lead to a significant increase of the A(0.236)/A(0.076) ratio^21^,^41^. Importantly, the prevalence of a specific G272S replacement (that also occurred in combination with S223N) increased over time. Interestingly, these aa substitutions are highly specific for Egyptian H5N1 viruses and not conserved or very rare in the HA proteins of viruses isolated in other parts of Africa or in Europe and America. To obtain further evidence for a potentially increased pandemic risk associated with H5N1 HPAIV carrying these mutations, we characterized the respective mutations in the structural context of an HA of an IAV strain from another geographical region. To this end, we introduced 10 mutations (alone and/or in combination) into the HA coding sequence of the prototype Asian strain A/Thailand/KAN-1/2004 (KAN-1, H5N1) and determined the A(0.236)/A(0.076) amplitude ratios (Supplementary Table S2). Among all the mutations tested, K153D, S223(N,I) and G272S were found to increase the amplitude at the frequency F(0.236) most profoundly, the latter representing the frequency determined for human-adapted IAV strains (Fig. 1). The amplitude increase at F(0.236) was linked to an amplitude reduction at F(0.076), indicating an improved interaction with the avian receptor. The A(0.236)/A(0.076) ratio was found to be increased from 0.77 to 1.26, suggesting an increased pandemic potential for viruses carrying these mutations (see above). As indicated above, the prevalence of the G272S mutation in H5N1-HA sequences isolated from virus strains circulating in Egypt has strongly increased since 2009, particularly in viruses isolated from humans (from 5.6% in 2009 to 95.7% in 2011, see Supplementary Table S1). Furthermore, the (only) two HA sequences determined in 2012 for Egyptian isolates of human HAPIV were shown to carry the double mutation G272S/S223N.

### Generation of H5-HA wildtype and mutant pseudotyped retroviral particles (PRP)

**Figure 2:**
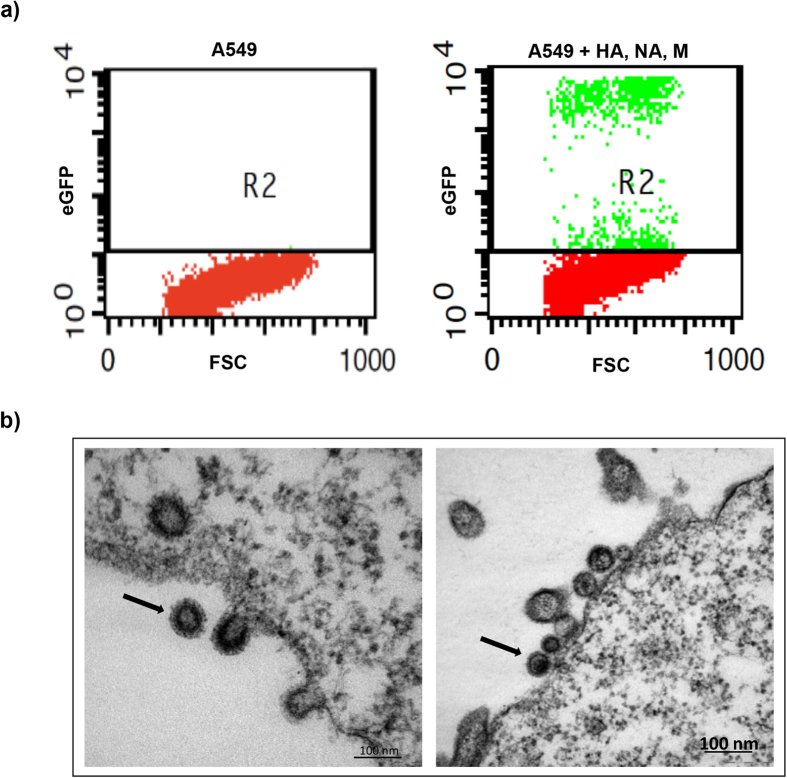
Characterization of HA-pseudotyped KAN-1 wildtype PRPs. **a**) FACS analysis of eGFP expression in A549 cells untransduced (left) or transduced (right) with wildtype HA/NA/M/eGFP pseudotyped retroviral particles. **b**) Electron microscopic images of wildtype H5N1 PRPs produced from 293T cells. A free immature PRP (left panel), as well as a mature PRP (right panel) are marked by an arrow. An electron lucent core characterizes the immature particle on the left panel, whereas the more mature particle on the right panel has an electron dense core.

To corroborate the predicted role of specific HA mutations in human receptor usage, we introduced the appropriate mutations into the KAN-1 HA sequence and studied potential effects on virus entry in two cell lines that either express avian or human receptors. We first generated retroviral particles pseudotyped with wildtype KAN-1/HA or mutant forms of HA carrying the mutations predicted by ISM to confer higher preference for α2,6-SA (Table 1). Pseudotyped retroviral particles (PRP) were generated from 293T cells transfected with a plasmid expressing wildtype and mutant H5-HA, respectively, and a set of plasmids expressing the NA and M proteins of KAN-1, MLV GagPol and the GFP marker protein, respectively. PRP infectivity was confirmed by FACS analysis of GFP expression after transduction of A549 cells with supernatants of transfected cells (Fig. 2a).Titers of the different wildtype and mutant PRP stocks determined on A549 cells varied between 2.4 × 10^4^/ml and 2.3 × 10^7^/ml (Table 1). Thin-section electron microscopy of transfected 293T producer cells revealed the expected morphology of immature and mature PRPs as well as budding structures (Fig. 2b). The retroviral particles displayed a dense envelope layer suggesting efficient expression and incorporation of the IAV surface proteins.

### Determination of the receptor specificity of mutated H5-HA PRP

**Figure 3:**
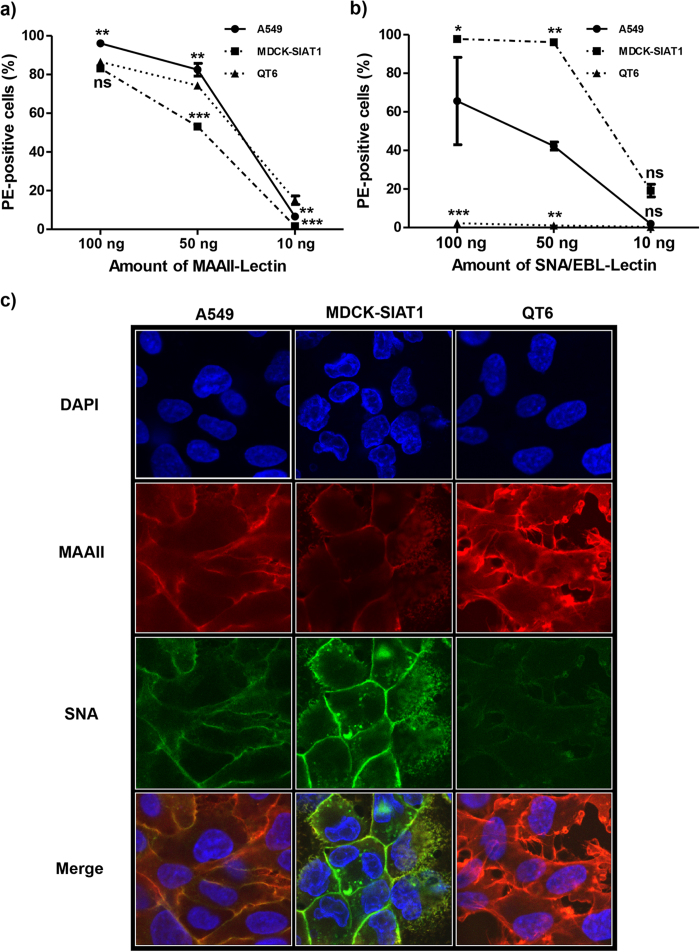
Determination of α2,6- and α2,3 receptors on MDCK-SIAT1, A549 and QT6 cells with specific lectins. **a,b)** The binding of varying amounts of biotinylated lectins MAAII (specific for avian α2,3 receptors) and SNA/EBL (specific for human α2,6 receptors) to QT6 and MDCK-SIAT1 cells as well as A549 control cells was determined by FACS analysis using streptavidine-PE. The differences of binding of varying amounts of biotinylated lectins to different cell lines were statistically compared to QT6 for MAAII-lectin binding or A549 for SNA/EBL-lectin binding using two-way ANOVA, followed by Bonferroni *post hoc* test. Significant differences between data sets are indicated by asterisks (*), whilst (ns) indicates non-significant differences. **c**) Formaldehyde-fixed monolayers of the indicated cell types were incubated with fluorescein-labelled SNA lectin to detect α2,6-linked sialic acids and biotinylated MAAII lectin and streptavidin-Cy3 to detect α2,3-linked sialic acids. The cellular membrane was permeabilized, the nuclei were stained with DAPI and analyzed using confocal microscopy.

To investigate if the HA mutations improve α2,6-SA receptor usage (as suggested by the ISM analysis, see above), we analyzed the infectivity of the corresponding mutant PRP on cells expressing different amounts of α2,3- and α2,6-SA (QT6, MDCK-SIAT1). QT6 is known to primarily express α2,3-SA (the avian IAV-type receptor), while MDCK-SIAT1 mainly expresses α2,6-SA (the human IAV-type receptor). Expression levels of the two receptors were determined by differential lectin-binding analysis (Fig. 3). Varying concentrations of biotinylated lectins, MAAII specific for α2,3-SA and SNA/EBL specific for α2,6-SA, were added to the cells and cell-bound lectins were quantified by FACS analysis using PE-labelled streptavidine. A549 cells, which were used to determine the titers of our PRP stocks, were included as control (Fig. 3).

As expected, both types of lectins were bound by A549 cells at high levels, indicating good expression of both receptor types (Fig. 3a,b). In contrast, QT6 cells only bound to MAAII lectin, whereas SNA/EBL binding was not detectable, not even at the highest lectin concentration used (100 ng) (Fig. 3b). MDCK-SIAT1 cells were shown to bind both types of lectins, indicating the presence of both receptors. However, the amount of α2,6-SA was significantly increased compared to A549 control cells (Fig. 3b). The differential expression of both receptors on these cell lines was confirmed by confocal laser microscopy (Fig. 3c). Based on these observations, we decided to use QT6 cells (expressing predominantly avian IAV receptors) and MDCK-SIAT1 cells (overexpressing human IAV receptors) in subsequent differential infectivity experiments.

**Figure 4:**
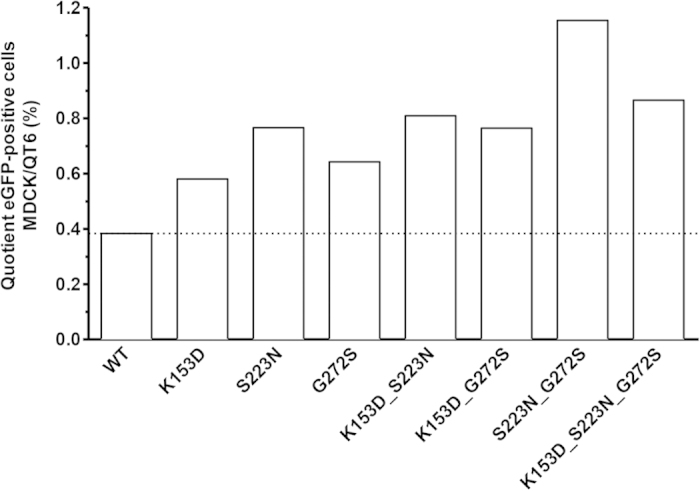
Infectivity of wildtype and HA-mutated PRPs on QT6 and MDCK-SIAT1 cells. Equal numbers of MDCK-SIAT and QT6 cells, respectively, were transduced with the indicated PRPs at an MOI of 0.2 and GFP-positive cells were counted by FACS analysis at 72 h post transduction. The ratio of GFP-positive MDCK-SIAT/QT6 cells is shown for the mutants compared to wildtype. Shown are the results of a representative experiment. The MDCK-SIAT/QT6 ratio was calculated from median values determined from triplicate cultures performed for each of the mutants.

Potential differences in receptor usage by individual mutant PRPs (compared to the wildtype PRP) were assessed by determining the ratio between GFP-positive MDCK-SIAT1 and GFP-positive QT6 cells following transduction with equal amounts of wildtype and mutant PRP, respectively. All mutants predicted by ISM to result in increased α2,6-SA usage showed better infectivity on MDCK-SIAT1 cells as reflected by increased MDCK-SIAT1/QT6 ratios compared to wildtype HA (Fig. 4). The double mutant S223N/G272S showed the highest MDCK-SIAT1/QT6 ratio, which was not further increased, but rather decreased, in the triple mutant K153D/S223N/G272S. Thus, the increased usage of human α2,6-SA receptors by the mutations in HA predicted by ISM was experimentally confirmed as reflected by better infectivity of the corresponding mutant PRP on cells overexpressing the human receptor.

### Replication kinetics of IAV HA mutants in MDCK-II cells

**Figure 5:**
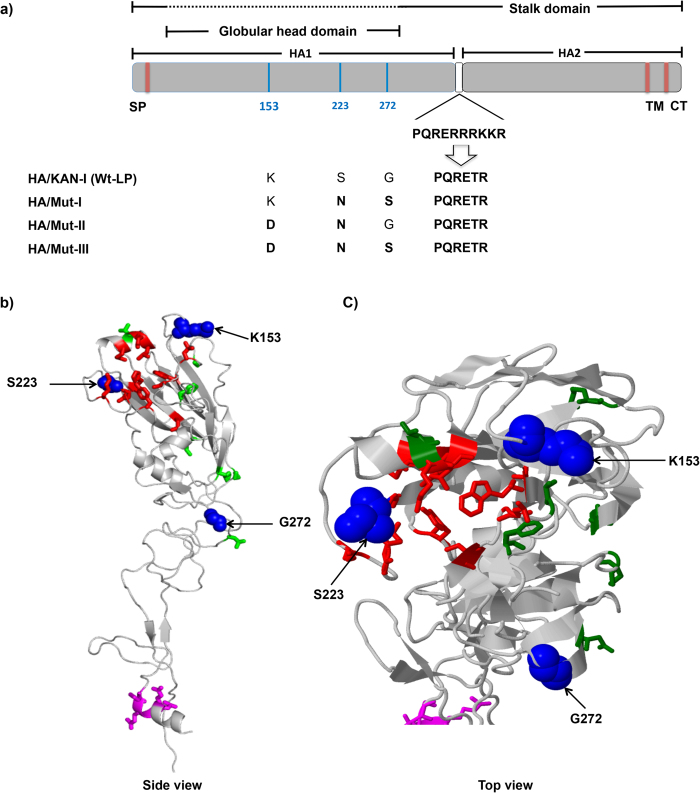
Location of the studied amino acid (aa) in the H5-HA of avian influenza A/Thailand/1(KAN-1)/2004 (H5N1) virus. **a**) The schematic representation shows the relative locations of H5-HA mutations in HA1 at aa positions 153, 223 and 272 (indicated in blue). The multibasic protease cleavage site present in the original KAN-1 isolate was removed in all recombinant viruses generated in this study (Wt-LP and Mut-I to -III). SP: signal peptide; TM: transmembrane domain; CT: cytoplasmic tail. **b**) and **c**) 3D structure model of H5 HA1 (for details, see Materials and Methods). Amino acid residues at positions 153, 223 and 272 (blue, spheres) are shown in side and top view in relation to the receptor-binding sites (red side chains). The other 7 aa that were previously identified by ISM in H5N1 viruses circulating in Egypt between 2006 and 2012, which also increase the A(0.236)/A(0.076) ratio (74, 110, 127, 143, 188, 234, 275), are shown as green side chains.

To assess potential effects of HA mutations on viral replication, we used reverse genetics to produce and characterize a set of IAV mutants based on a genetically modified form of KAN-1 (designated Wt-LP) that (for biological safety reasons) lacked a multi-basic cleavage site in its HA protein (Fig. 5a). The mutant viruses derived from the parental Wt-LP virus carried the HA mutations that had been shown to cause the strongest effects in our PRP infectivity assays (see above) (M-I, M-II and M-III). Figure 5b,c indicate the location (in the globular head domain, HA1, of the molecule) of the three aa residues 153, 223 and 272 that we have analyzed.

**Figure 6:**
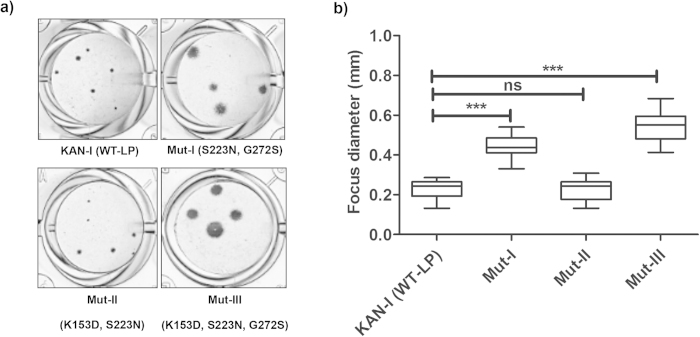
Focus phenotypes of recombinant KAN-1 (Wt-LP) and H5-HA mutant KAN-1 viruses on MDCK cells. **a**) Focus assay showing different focus sizes. MDCK-II cells were infected with the parental and mutated viruses at an MOI of 0.001 for 24 h and then stained using a monoclonal antibody against NP. **b**) Average focus sizes were determined from 50 foci for each virus. Asterisks (*) indicate a significant difference (p < 0.05) compared to the parental KAN-1 (Wt-LP) virus, while (ns) indicates non-significant difference. Statistical analysis was performed using one-way ANOVA, followed by Turkey’s *post hoc* test.

Focus size and morphology were analyzed using MDCK-II cells (Fig. 6a) and the relative distribution of focus diameters was determined (Fig. 6b). The M-II virus (K153D, S223N) produced foci similar in size to those produced by the parental WT-LP virus, while foci produced by the M-I (S223N, G272S) and M-III (K153D, S223N, G272S) mutants were significantly larger than those of KAN-1 (Wt-LP). This indicates that the M-II and -III viruses have a propagation advantage in MDCK-II cells compared to the parental virus.

### Replication of H5N1 IAV mutants in different cell types *in vitro*

**Figure 7:**
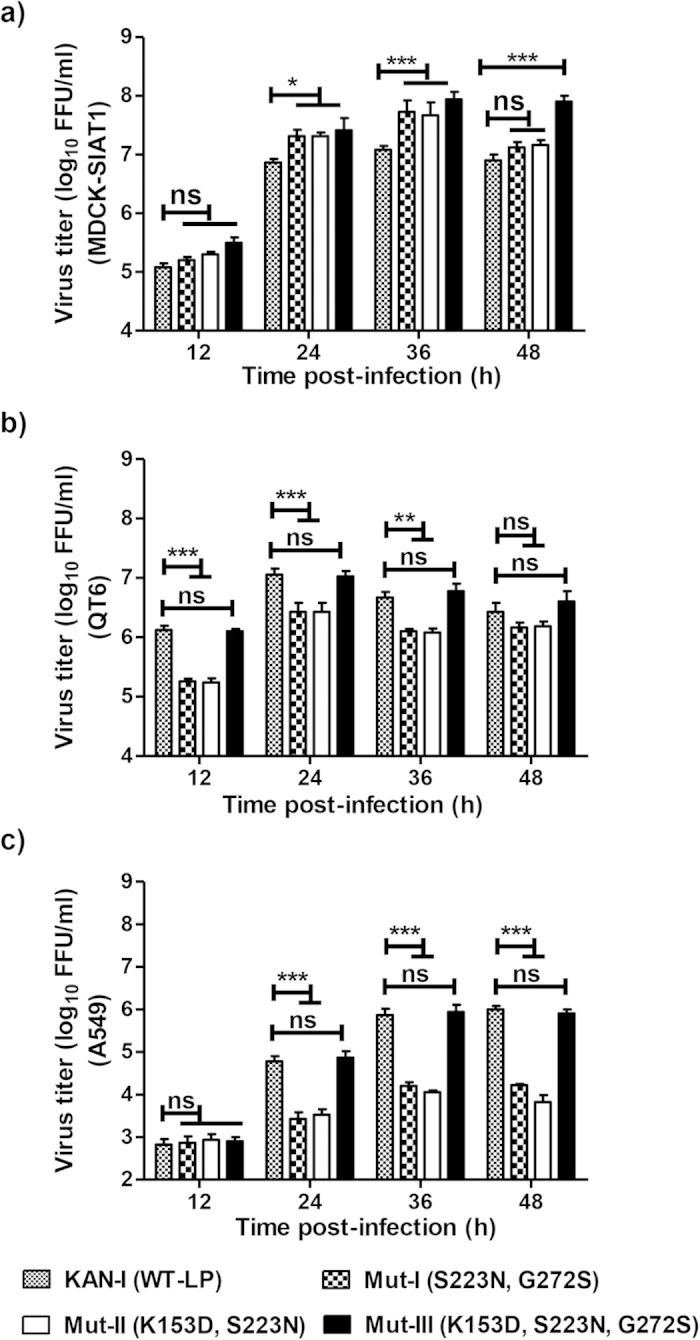
*In vitro* growth kinetics of KAN-1 (Wt-LP) and H5-HA mutant KAN-1 viruses. **a**) MDCK-SIAT1, **b**) QT6, or **c**) A549 cells were infected at an MOI of 0.001. The infectious virus and HA titers were determined at the indicated time points in MDCK-II cells by focus assay (FFU/ml) and standardized hemagglutination assay (HAU/ml). Mean values of results represent the averages from three independent experiments and are presented with standard deviations (SDs) indicated by error bars. Asterisks (*) indicate a significant difference (p < 0.05) compared to the parental KAN-1 (Wt-LP) virus while (ns) indicates a non-significant difference. Statistical analysis was performed using repeated measures ANOVA, followed by Bonferroni *post hoc* test.

The potential impact of the introduced HA mutations on viral replication was further investigated in (i) MDCK-SIAT1 cells, which overexpress α2,6-SA^25^, (ii) QT6 cells, which overexpress α2,3-SA, and (iii) A549 cells, which express similar amounts of α2,3-SA and α2,6-SA (Fig. 3). Confluent cell monolayers were infected with parental or mutant viruses at an MOI of 0.001 FFU/cell and maintained in trypsin-containing cell culture medium. Virus titers in supernatants collected at 12, 24, 36 and 48 h p.i. were determined as FFU/ml on MDCK-II cells^40^. In MDCK-SIAT1 cells (Fig. 7a), the titersof all mutants were approximately 0.5 log_10_ FFU/ml higher than that of the KAN-1 (Wt-LP) at 24–36 h p.i. However, at 48 h p.i., the M-III virus showed a significant increase in titer of about 1.0 log_10_ FFU/ml compared to both the parental KAN-1 (Wt-LP) and the other two mutants (Fig. 7a). Conversely, in QT6 (Fig. 7b) and A549 (Fig. 7c) cells, the parental KAN-1 (Wt-LP) and M-III viruses replicated to similar titers with no apparent differences in infectious particle number. In contrast, M-I and -II virus titers were significantly reduced by 0.5–1.0 log_10_ FFU/ml compared to the parental KAN-1 (Wt-LP) virus at 24–48 h p.i (Fig. 7b,c). Taken together, these results suggest that the triple mutation in HA (K153D/S223N/G272S; Mut-III) enhances viral replication in cells that overexpress α2,6-SA (MDCK-SIAT1), while there was no such difference in A549 and QT6 cells, which express low(er) amounts of α2,6-SA (compared to MDCK-SIAT1). The double mutants, S223N/G272S and K153D/S223N (Mut-I, Mut-II), also replicated more efficiently in MDCK-SIAT1 cells. However, in this case, the increase was much less profound compared to the triple mutant. In A549 and QT6 cells, the double mutants replicated to lower titers compared to both the parental virus and the triple mutant, possibly indicating that the binding to α2,3-SA was reduced in these mutants.

## Discussion

H5N1-type HPAIV are associated with high mortality in humans (>60%) and therefore represent a serious threat to human health. WHO launched extensive surveillance and monitoring programs for IAV^42^ and the development of new tools for early detection of new strains and prevention of IAV pandemics is of high priority. To date, H5N1 viruses have not (yet) been transmitted efficiently among humans, but there is a risk of this host restriction being overcome by reassortment of genome segments between H5N1 and co-circulating human H1N1 or H3N2 IAV strains.

Recent studies suggest that H5N1 HPAIV isolated in Egypt over the past few years increasingly exhibit a receptor usage that is similar to that of seasonal human H1N1 viruses, possibly indicating more efficient human-to-human transmission and increased pandemic potential in the (near) future^24^,^43^. An antigenic cartography of viruses isolated in Egypt revealed 6 distinct antigenic groups^44^. In line with our data and conclusions presented above, 19 of the 27 mutations presumably involved in adaptation of H5N1 viruses to the human receptor are associated with an increase of the A(0.236)/A(0.076) ratio^21^. Others possibly increase the affinity of the HA to the human receptor by a better adaptation of the receptor binding domain (RBD).

In this study, we used the ISM bioinformatic tool to characterize a set of mutations in the H5-HA that likely increase the pandemic potential of a prototype HPAIV (KAN-1) strain. We found that the mutations, when introduced *in silico* into the wildtype KAN-1 HA sequence, led to an increase of the amplitude ratio A(0.236)/A(0.076) at the frequencies F at 0.236 and 0.076, the latter indicating critical interactions of HA with human and avian receptors, respectively (Fig. 1). The ratio increased from 0.77 to 1.26, with values above 1 suggesting increased human tropism^24^. By comparison, the amplitude ratio increased only from 0.72 to 0.96 (Supplementary Fig. S1) if substitutions known to increase IAV transmission by respiratory droplets in ferrets (N154D, Q222L, N220K, and T315I) were analyzed in the context of the HA protein of A/Vietnam/1203/2004 (H5N1)^45^.

In our ISM studies, the HA aa substitutions K153D, S223(N,I) and G272S (alone or in different combinations) increased the amplitude that indicates human receptor usage most profoundly (data not shown). Importantly, S223N and, particularly, G272S have also been detected in H5N1 HPAIVs circulating in Egypt and the number of viruses carrying these mutations has significantly increased since 2008 (Supplementary Table S1), correlating with an increasing number of human H5N1 infections in this country. The fact that the number of human cases in Egypt did not increase more dramatically in recent years might reflect a need for further adaptive requirements, for example in the receptor binding domain. Interestingly, S223N substitutions were only detected in human samples and combinations of the two aa substitutions were detected in both human H5N1 HPAIV HA sequences reported in 2012, further supporting the significance of these aa substitutions in viral adaptation to human hosts and suggesting an increased pandemic potential for H5N1 HPAIV strains currently circulating in Egypt (Supplementary Table S1).

Substitutions at position 153 (150-Loop) and 223 (220-Loop) of the receptor-binding site in the globular head domain of H5-HA are expected to affect the structure of the receptor-binding site and, thus, may influence receptor specificity. Amino acid mutations K153R and S223N/G were shown to alter the antigenic properties and/or receptor specificity^34^,^46^,^47^,^48^. However, it is currently not clear if the recently reported adaptive substitution G272S in the H5-HA protein of Egyptian isolates has similar effects on the receptor specificity^20^. In a recent attempt to establish a model that predicts the evolution of the viral population, the analysis of 3,944 HA sequences from H3N2 IAV revealed that adaptive mutations, which influence biological and immunological properties of these viruses, occur with highest probability within the epitopes A–D^49^. The alterations at the three selected positions (153, 223, 272) investigated here are also located within epitopes A, B and C, suggesting that with high probability such variations can be expected to occur also in H5-type HA of HPAIV as a consequence of natural viral adaptation.

To provide more direct evidence for an involvement of specific HA aa residues in the adaptation to human receptor usage we made appropriate replacements (predicted by ISM to alter the amplitude ratio A(0.236)/A(0.076) towards human receptor usage) in the HA protein of the A/Thailand/KAN-1/2004 (KAN-1) strain. Single or combined replacements of K153D, S223N and G272S were introduced into the H5-HA of KAN-1. Potential effects of the introduced aa substitutions were studied by using (i) retroviral particles pseudotyped with either the wildytpe or the respective mutant HAs and (ii) recombinant viruses based on an attenuated KAN-1 virus (WT-LP).

Transduction of PRPs expressing GFP and a wildtype or mutant form of HA into MDCK-SIAT1 cells (overexpressing the human receptor type) and QT6 cells (with very low expression of the human receptor type) revealed an increased MDCK-SIAT1/QT6 ratio for all these mutants compared to wildtype (Fig. 4), strongly supporting our ISM-based predictions on more efficient human receptor usage for these mutants. Notably, ISM analyses of double and triple mutations consistently showed a decrease in amplitude at the frequency for avian receptor usage (0.076) accompanied by an increase at the frequency for human receptor usage (0.236). This was more pronounced for the triple mutation (Supplementary Table S2). Even though the triple mutations substantially decreased the amplitude of the avian frequency compared to wildtype (Supplementary Table S2), the results shown in Fig. 7B indicate no decrease of viral replication in QT6 cells. We think that combinations of mutations do not only affect long-range interaction properties of HA1 but may also affect its 3D structure. For this reason, the triple mutation (even though it increases the calculated efficacy of recognition and targeting between HA1 and the human receptor) might not necessarily disturb the chemical binding to the avian receptor.

The PRP study was complemented by the characterization of recombinant viruses derived from KAN-1 (Wt-LP) that express different mutant forms of HA with combined replacements at positions 153, 223 and 272 (K153D, S223N and G272S). The data show that mutants containing combined S223N and G272S replacements produce larger foci in mammalian MDCK cells (Fig. 6). Compared to the parental KAN-1 (Wt-LP) virus, viruses containing double or triple replacements replicated significantly more efficiently in MDCK-SIAT1 cells overexpressing α2,6-SA (as determined by FFU/ml). In contrast, differential growth kinetics of wt versus HA mutant viruses was not observed in cells expressing only the avian HA receptor type, indicating that these changes do not represent a propagation disadvantage in avian hosts.

Recent human infections with a low pathogenic avian influenza virus (LPAIV) of the H7N9-type have initiated a paradigm change^50^,^51^. Previously, studies into the pandemic potential for humans were largely focused on HPAIV of the H5N1 and H7N3/H7N7 subtypes. While H5N1 HPAIV are known to cause severe disease in humans and many birds, the H7-type is generally associated with mild disease in humans but causes fatal disease in a wide range of birds including economically relevant species (chicken, turkeys, etc.)^52^,^53^. However, recent work shows that also specific H7-type HPAIV strains have the potential to replicate in mammalian species^40^,^54^. These observations have led to more extensive research and monitoring efforts to better understand the specific pathogenic and epidemiological characteristics of these HPAIV. Additionally, the large group of LPAIV and the recently documented H7N9, H10N8 and H6N1 LPAIV cases in humans extend the list of subtypes potentially causing human disease and thus deserve further studies^55^,^56^. The increasing number of cases with IAV subtypes not considered previously to cause disease in humans highlights the need to develop new tools (including *in silico* methods) that are suitable to closely monitor the biological properties (tropism, pathogenic potential in specific species, etc.) of circulating IAV strains and identify mutations indicative of CPIV. These tools might help the health authorities, healthcare systems, and vaccine manufacturers to plan ahead and respond earlier to newly emerging IAVs with pandemic potential. The bioinformatic method used in the present study to define mutations in H5N1 IAV that indicate viral adaptation to humans and thus increase the pandemic potential of these viruses may be a suitable tool to identify CPIV among circulating influenza viruses in different populations.

## Additional Information

**How to cite this article**: Schmier, S. *et al.* *In Silico* Prediction and Experimental Confirmation of HA Residues Conferring Enhanced Human Receptor Specificity of H5N1 Influenza A Viruses. *Sci. Rep.* **5**, 11434; doi: 10.1038/srep11434 (2015).

## Supplementary Material

Supplementary Information

## Figures and Tables

**Table 1:** Infectious titers of the different HA pseudotyped retroviral particles on A549 cells.

WT	5.1 × 10^5^/ml
K153D	2.2 × 10^6^/ml
S223N	8.3 × 10^6^/ml
G272S	3.5 × 10^6^/ml
K153D/S223N	2.4 × 10^4^/ml
K153D/G272S	not analyzed
S223N/G272S	2.3 × 10^7^/ml
K153D/S223N/G272S	4.3 × 10^5^/ml
