# Supplementary material for: In Silico Prediction and Experimental Confirmation of HA Residues Conferring Enhanced Human Receptor Specificity of H5N1 Influenza A Viruses
Source: Sci Rep. 2015 Jun 19;5:11434. doi: 10.1038/srep11434 (PMC4473683; doi:10.1038/srep11434)
Supplement: Supplementary Information [file srep11434-s1.pdf]

In Silico Prediction and Experimental Confirmation of HA Residues Conferring Enhanced Human Receptor Specificity of H5N1 Influenza A Viruses

Sonja Schmier, Ahmed Mostafa, Thomas Haarmann, Norbert Bannert,

John Ziebuhr , Veljko Veljkovic, Ursula Dietrich, Stephan Pleschka

Supplementary Table S1:

Presence of mutations S223N and G272S in avian/human HA1 sequences from H5N1 viruses isolated in Egypt in the period 2006-2012

**year**	**G272S**		**S223N***	**G272S + S223N**	
	avian/human	only human		avian/human	only human
2006	0/57	0/17	1/57 (1.8)*	0/57 (0)	0/17
2007	0/134	0/26	1/134 (0.8)*	0/134 (0)	0/26
2008	0/122	0/8	1/122 (0.8)*	0/122 (0)	0/8
2009	6/142 (4.2)	**2/36 (5.6)**	1/142 (0.7)*	0/142 (0)	0/36
2010	12/176 (6.8)	**4/17 (23.5)**	0/176 (0)	0/176 (0)	0/17
2011	61/145 (42.0)	**22/23 (95.7)**	1/145 (0.7)*	0/145 (0)	0/23
2012	31/32 (98.0)	**2/2 (100)**	1/32 (3.1)*	2/32 (6.2)	**2/2 (100)**

Numbers in parentheses indicate %. The increase in G272S and the double mutation in human H5N1 HA over time is marked in bold. *The six S223N replacements have all been identified in human IAV HA sequences.

Supplementary Table S2:

Effect of the combined mutations on amplitudes at the frequencies F(0.076) and F(0.236) corresponding to interactions with avian and human receptors

Mutations	AF(0.076)	AF(0.236)	AF(0.236)/AF(0.076)
Wt	3.47	2.68	0.77
K153D	3.14	2.98	0.95
S223N	3.34	2.95	0.88
G272S	3.20	2.89	0.90
K153D+S223N	3.01	3.26	1.08
K153D+G272S	2.89	3.20	1.11
S223N+G272S	3.07	3.17	1.03
K153D+S223N+G272S	2.77	3.49	1.26

HA1 sequences of the influenza virus A/Thailand/KAN-1/2004 (H5N1) and its mutants

A/Thailand/KAN-1/2004 (Wt)
DQICIGYHANNSTEQVDTIMEKNVTVTHAQDILEKTHNGKLCDLDGVKPLILRDCSVAGWLL
GNPMCDEFINVPEWSYIVEKANPVNDLCYPGDFNDYEELKHLLSRINHFEKIQIIPKSSWSS
HEASLGVSSACPYQRKSSFFRNVVWLIKKNSTYPTIKRSYNNTNQEDLLVLWGIHHPNDAAE
QTKLYQNPTTYISVGTSTLNQRLVPRIATRSKVNGQSGRMEFFWTILKPNDAINFESNGNFI
APEYAYKIVKKGDSTIMKSELEYGNCNTKCQTPMGAINSSMPFHNIHPLTIGECPKYVKSNR
LVLATGLRNSPQRERRRKKR

A/Thailand/KAN-1/2004 (K153D)
DQICIGYHANNSTEQVDTIMEKNVTVTHAQDILEKTHNGKLCDLDGVKPLILRDCSVAGWLL
GNPMCDEFINVPEWSYIVEKANPVNDLCYPGDFNDYEELKHLLSRINHFEKIQIIPKSSWSS
HEASLGVSSACPYQRKSSFFRNVVWLIK**D**NSTYPTIKRSYNNTNQEDLLVLWGIHHPNDAAE
QTKLYQNPTTYISVGTSTLNQRLVPRIATRSKVNGQSGRMEFFWTILKPNDAINFESNGNFI
APEYAYKIVKKGDSTIMKSELEYGNCNTKCQTPMGAINSSMPFHNIHPLTIGECPKYVKSNR
LVLATGLRNSPQRERRRKKR

A/Thailand/KAN-1/2004 (S223N)
DQICIGYHANNSTEQVDTIMEKNVTVTHAQDILEKTHNGKLCDLDGVKPLILRDCSVAGWLL
GNPMCDEFINVPEWSYIVEKANPVNDLCYPGDFNDYEELKHLLSRINHFEKIQIIPKSSWSS
HEASLGVSSACPYQRKSSFFRNVVWLIKKNSTYPTIKRSYNNTNQEDLLVLWGIHHPNDAAE
QTKLYQNPTTYISVGTSTLNQRLVPRIATRSKVNGQ**N**GRMEFFWTILKPNDAINFESNGNFI
APEYAYKIVKKGDSTIMKSELEYGNCNTKCQTPMGAINSSMPFHNIHPLTIGECPKYVKSNR
LVLATGLRNSPQRERRRKKR

A/Thailand/KAN-1/2004 (G272S)
DQICIGYHANNSTEQVDTIMEKNVTVTHAQDILEKTHNGKLCDLDGVKPLILRDCSVAGWLL
GNPMCDEFINVPEWSYIVEKANPVNDLCYPGDFNDYEELKHLLSRINHFEKIQIIPKSSWSS
HEASLGVSSACPYQRKSSFFRNVVWLIKKNSTYPTIKRSYNNTNQEDLLVLWGIHHPNDAAE
QTKLYQNPTTYISVGTSTLNQRLVPRIATRSKVNGQSGRMEFFWTILKPNDAINFESNGNFI
APEYAYKIVKKGDSTIMKSELEY**S**NCNTKCQTPMGAINSSMPFHNIHPLTIGECPKYVKSNR
LVLATGLRNSPQRERRRKKR

A/Thailand/KAN-1/2004 (K153D+S223N)
DQICIGYHANNSTEQVDTIMEKNVTVTHAQDILEKTHNGKLCDLDGVKPLILRDCSVAGWLL
GNPMCDEFINVPEWSYIVEKANPVNDLCYPGDFNDYEELKHLLSRINHFEKIQIIPKSSWSS
HEASLGVSSACPYQRKSSFFRNVVWLIK**D**NSTYPTIKRSYNNTNQEDLLVLWGIHHPNDAAE

QTKLYQNPTTYISVGTSTLNQRLVPRIATRSKVNGQ**N**GRMEFFWTILKPNDAINFESNGNFI
APEYAYKIVKKGDSTIMKSELEYGNCNTKCQTPMGAINSSMPFHNIHPLTIGECPKYVKSNR
LVLATGLRNSPQRERRRKKR

A/Thailand/KAN-1/2004 (K153D+G272S)

DQICIGYHANNSTEQVDTIMEKNVTVTHAQDILEKTHNGKLCDLDGVKPLILRDCSVAGWLL
GNPMCDEFINVPEWSYIVEKANPVNDLCYPGDFNDYEELKHLLSRINHFEKIQIIPKSSWSS
HEASLGVSSACPYQRKSSFFRNVVWLIK**D**NSTYPTIKRSYNNTNQEDLLVLWGIHHPNDAAE
QTKLYQNPTTYISVGTSTLNQRLVPRIATRSKVNGQSGRMEFFWTILKPNDAINFESNGNFI
APEYAYKIVKKGDSTIMKSELEY**S**NCNTKCQTPMGAINSSMPFHNIHPLTIGECPKYVKSNR
LVLATGLRNSPQRERRRKKR

A/Thailand/KAN-1/2004 (S223N+G272S)

DQICIGYHANNSTEQVDTIMEKNVTVTHAQDILEKTHNGKLCDLDGVKPLILRDCSVAGWLL
GNPMCDEFINVPEWSYIVEKANPVNDLCYPGDFNDYEELKHLLSRINHFEKIQIIPKSSWSS
HEASLGVSSACPYQRKSSFFRNVVWLIKKNSTYPTIKRSYNNTNQEDLLVLWGIHHPNDAAE
QTKLYQNPTTYISVGTSTLNQRLVPRIATRSKVNGQ**N**GRMEFFWTILKPNDAINFESNGNFI
APEYAYKIVKKGDSTIMKSELEY**S**NCNTKCQTPMGAINSSMPFHNIHPLTIGECPKYVKSNR
LVLATGLRNSPQRERRRKKR

A/Thailand/KAN-1/2004 (K153D+S223N+G272S)

DQICIGYHANNSTEQVDTIMEKNVTVTHAQDILEKTHNGKLCDLDGVKPLILRDCSVAGWLL
GNPMCDEFINVPEWSYIVEKANPVNDLCYPGDFNDYEELKHLLSRINHFEKIQIIPKSSWSS
HEASLGVSSACPYQRKSSFFRNVVWLIK**D**NSTYPTIKRSYNNTNQEDLLVLWGIHHPNDAAE
QTKLYQNPTTYISVGTSTLNQRLVPRIATRSKVNGQ**N**GRMEFFWTILKPNDAINFESNGNFI
APEYAYKIVKKGDSTIMKSELEY**S**NCNTKCQTPMGAINSSMPFHNIHPLTIGECPKYVKSNR
LVLATGLRNSPQRERRRKKR

Supplementary Figure S1: Effect of mutations N154D, Q222L, N220K and T315I on the informational spectrum (IS) of HA from H5N1 virus A/Vietnam/1203/2004 (H5N1).

A) IS of wildtype HA. **B)** IS of HA with combined mutations. Frequencies at 0.076 and 0.236 for avian and human receptor specificity are indicated.

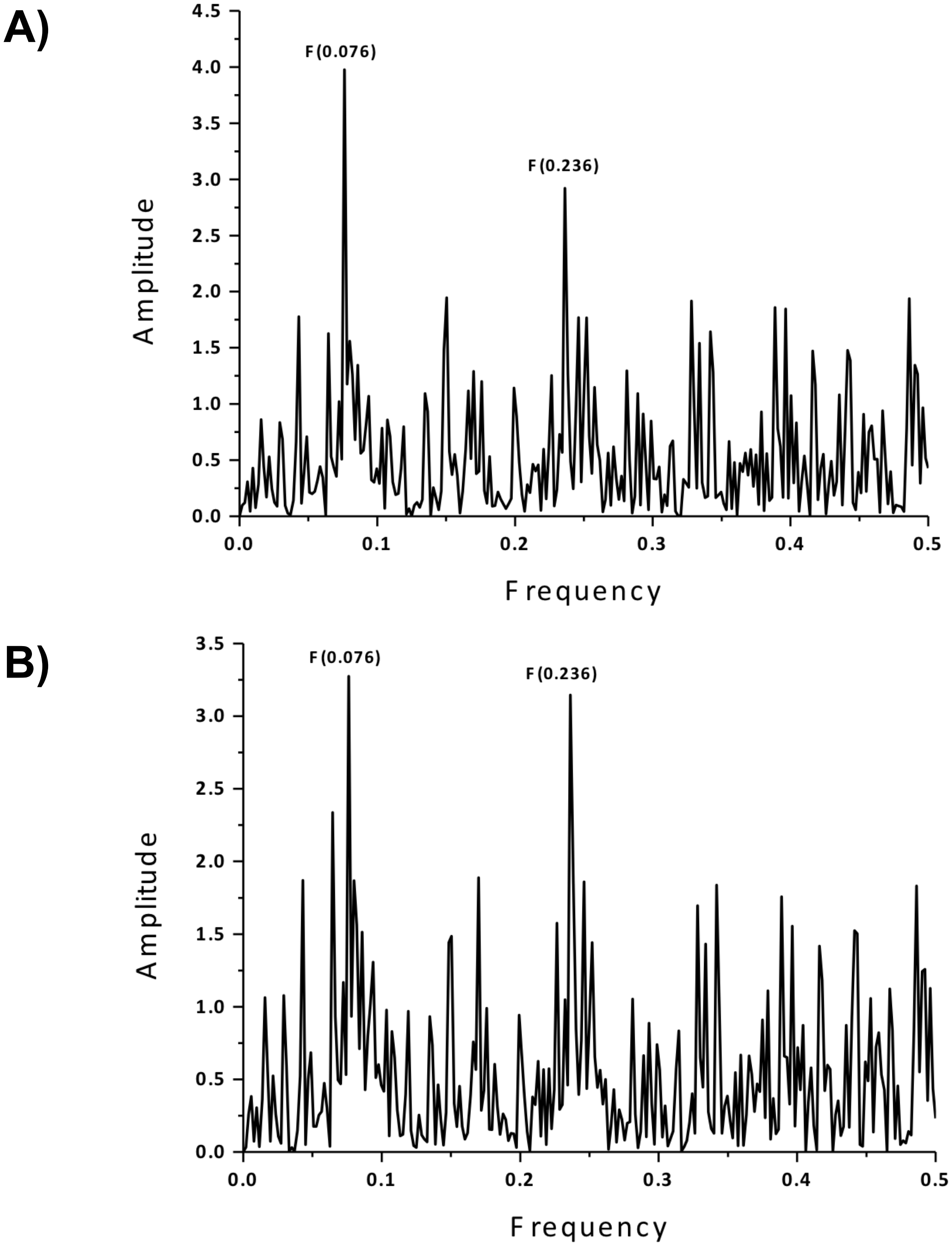
A)
F(0.076)
F(0.236)
Amplitude
Frequency
B)
F(0.076)
F(0.236)
Amplitude
Frequency
